# Meat Inspection at the Munich Abattoir*Wochenschrift für Thierheilkunde, No. 19, 1884.

**Published:** 1884-10

**Authors:** 


MEAT INSPECTION AT THE MUNICH ABATTOIR.*—Four thousand, six hundred and twenty-four animals were condemned for disease during the year 1883: 363 being oxen, 1809 cows, 156 steers, 59 young cattle, 883 calves; 301 swine; 957 sheep; 28 horses, and 68 smaller animals: 1295 *of these were tuberculous*, viz., 235 oxen, 980 cows, 47 steers, and 33 young cattle; 2.63 per cent. Fifteen swine were also condemned on account of tuberculosis; 43 oxen, 48 cows, 9 steers, and 2 young cattle were condemned for foot and mouth disease; 620 cases of indurated liver, 620 of distoma disease, 232 of echinococcus, 54 of pulmonary abscess, 15 with kidney disease, were also re-

* Wochenschrift für Thierheilkunde, No. 19, 1884.

ported. Of the hogs, 90 were condemned for measles, and 33 for hog cholera.

